# *Emayella augustorita* [em″-ǝ-yel′-ǝ aw-goost′-ō-rē″-tǝ]

**DOI:** 10.3201/eid3105.240723

**Published:** 2025-05

**Authors:** Clyde Partin

**Affiliations:** Emory University School of Medicine, Atlanta, Georgia, USA

**Keywords:** Emayella augustorita, bacteria, Pasteurellaceae, gram-negative, commensal bacteria, zoonoses, cats, dogs, Limoges, France

In 2024, a novel bacterial genus and species of the Pasteurellaceae family, *Emayella augustorita*, was presented by a team led by Sylvain Meyer from the University of Limoges in Limoges, France. *E. augustorita* is a fermentative, gram-negative organism and a commensal and common inhabitant of feline and canine oral cavities and upper respiratory tracts (Figure). This newly identified rod-shaped bacterium was isolated from blood cultures in a woman who had sepsis because of an infected metallic biliary stent.

**Figure:**
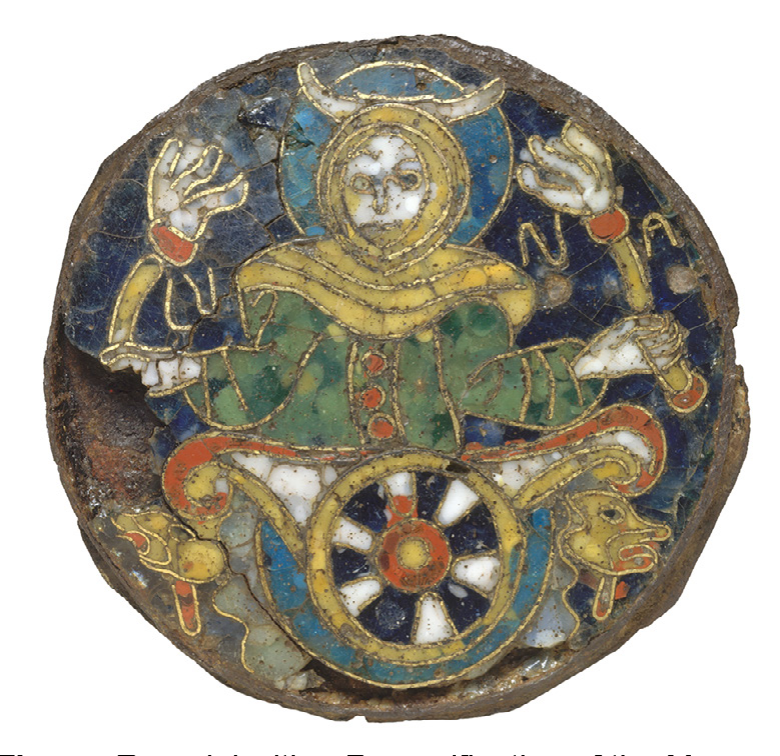
Roundel with a Personification of the Moon, ca. 860–890. This cloisonné-enamel plaque, made of copper alloy with an iron back plate, was created in south-central France, in the area of what is now Limoges. Dimensions: 3 3/8 x 1/4 in (8.6 x 0.6 cm). Public domain image courtesy of The Metropolitan Museum of Art (New York, NY, USA).

As a nod to local heritage, in naming their discovery, the authors chose *Emayella*, which translates from Latin as enamel. Limoges is known for its artistic heritage, especially for enamel-painted metalwork dating to the Middle Ages. In the 18th Century, after a kaolin pit was discovered nearby, Limoges became an avatar of exquisite porcelain ware. The species epithet, *augustorita*, in honor of the Emperor Augustus, was the original name given to the town by its Roman founders, in 10 BCE. The Gaulish suffix -*ritum* (*rito* or ford) is a reference to the city’s location on a ford on the Vienne River. The name Limoges itself evolved from a Latin word, Lemovices, referring to a Gaulish tribe who armed themselves with spears fashioned from elm.
